# Machine-learning approach to the design of OSDAs for zeolite beta

**DOI:** 10.1073/pnas.1818763116

**Published:** 2019-02-07

**Authors:** Frits Daeyaert, Fengdan Ye, Michael W. Deem

**Affiliations:** ^a^Department of Bioengineering, Rice University, Houston, TX 77005;; ^b^Department of Physics & Astronomy, Rice University, Houston, TX 77005

**Keywords:** machine learning, neural network, zeolite beta, OSDA

## Abstract

We report a machine-learning strategy for design of organic structure directing agents (OSDAs) for zeolite beta. We use machine learning to replace a computationally expensive molecular dynamics evaluation of the stabilization energy of the OSDA inside zeolite beta with a neural network prediction. We train the neural network on 4,781 candidate OSDAs, spanning a range of stabilization energies. We find that the stabilization energies predicted by the neural network are highly correlated with the molecular dynamics computations. We further find that the evolutionary design algorithm samples the space of chemically feasible OSDAs thoroughly. In total, we find 469 OSDAs with verified stabilization energies below −17 kJ/(mol Si), comparable to or better than known OSDAs for zeolite beta, and greatly expanding our previous list of 152 such predicted OSDAs. We expect that these OSDAs will lead to syntheses of zeolite beta.

## Significance

Zeolite beta is one of the top-six zeolites of commercial interest. It has been synthesized through the use of a number of organic structure directing agents (OSDAs). Pure zeolite beta A has not yet been synthesized, nor has chiral zeolite beta A. We here report a machine-learning strategy to aid the computational design of chemically synthesizable OSDAs for zeolite beta A. The use of machine learning speeds up the computation by a factor of 350. Through de novo materials design runs, a total of 3,062 promising OSDAs were identified, and 469 OSDAs were computed to stabilize the structure of zeolite beta A better than known compounds.

Zeolites are crystalline nanoporous aluminosilicate minerals that have wide use in absorption, separation, and catalysis (1). Presently, a total of 245 zeolite structures, both natural and man-made and differing in structure and pore size, have been identified (2). Zeolite beta is a large 3D 12-ring channel system (3), and it is one of the 17 zeolites of commercial interest (4). Its industrial uses include the alkylation of benzene (5) and the separation of organics from water (6). Synthetic zeolites such as zeolite beta are synthesized by hydrothermal synthesis from suitable amorphous aluminosilicate precursors (7). To direct the synthesis toward a particular zeolite structure, organic bases that act as templates, termed organic structure directing agents (OSDAs), are added to the reaction medium (8, 9). While template-free syntheses of zeolite beta have been reported (10), the main synthetic route uses tetraethyl amine as the OSDA (11). Syntheses of zeolite beta with roughly 50–100 other OSDAs have been reported.

Zeolite beta consists of three polymorphs: polymorph A (BEA), polymorph B (BEB), and polymorph C (BEC) (3). At present, no synthetic route to pure BEA has been obtained. Existing formulations of zeolite beta lead to an intergrown hybrid structure of BEA and BEB (3). Uniformly structured zeolites can lead to smaller, cleaner, and more efficient catalytic processes (12). Moreover, the BEA polymorph is chiral, and an enantiomerically enriched form of pure BEA would be of great interest for enantiospecific catalysis and separation (13). Ongoing research in our group is therefore directed toward the design of suitable OSDAs leading to both pure and enantiomerically enriched BEA.

Selectivity toward a given zeolite is promoted by a structure directing agent and depends to a large degree on favorable nonbonding interactions governed by packing in the zeolite framework (14). In the past, we have successfully built upon this observation to use structure-based molecular design to obtain OSDAs for several zeolites (15–17), including a chiral OSDA leading to an enantiomerically enriched zeolite STW (18). The methods we have applied in these efforts include algorithms both for de novo design (19, 20) and virtual combinatorial chemistry (21), as well as virtual screening of selected sets of available compounds. At the heart of these algorithms is a computational procedure to predict the suitability of a molecule to serve as OSDA for a given zeolite (19). The scoring function calculates a series of molecular properties of increasing computational complexity, with the least computationally intensive properties being used as filters (22). The most computationally intensive calculation consists of a molecular dynamics evaluation of the stabilization energy of a putative OSDA in the target zeolite and requires on the order of 3 h of CPU time when the target is BEA. A de novo design or virtual combinatorial chemistry experiment typically requires on the order of 200,000 calls of the scoring function, of which around 10% reach the stage of the molecular dynamics run. In view of our efforts to design OSDAs for zeolite BEA, it is of great interest to us to speed up the evaluation of this scoring function. In our research so far we have performed a large number of calculations, and in this paper we describe our efforts to effectively tap this database of information using a data-driven approach.

Machine-learning (ML) algorithms that synthesize existing data to produce predictive models are seeing a revival in molecular and materials science thanks to the growing availability of massive numbers of data (23). Examples include algorithms for quantum chemistry (24), retrosynthetic chemistry (25), and de novo design (26, 27). Once properly trained, an ML algorithm is very fast to produce an output from new input. Therefore, given the large number of predicted stabilization energies of putative BEA OSDAs that we have collected thus far, we have trained an ML algorithm to build a quantitative structure–property relationship to accurately and efficiently predict OSDA-BEA stabilization energies. That is, we trained neural networks to predict OSDA stabilization energies based on their molecular structures. We have used 3D-MoRSE (Molecule Representation of Structures based on Electron diffraction) descriptors for the OSDA molecules (28, 29). These descriptors are input to the neural network as described in *Methods*. We used this ML approach to replace the molecular dynamics evaluation of the stabilization energy with a trained neural network. We further used this approach to produce putative BEA OSDAs.

## Results and Discussion

### Training the Models.

**Fig. 1.**
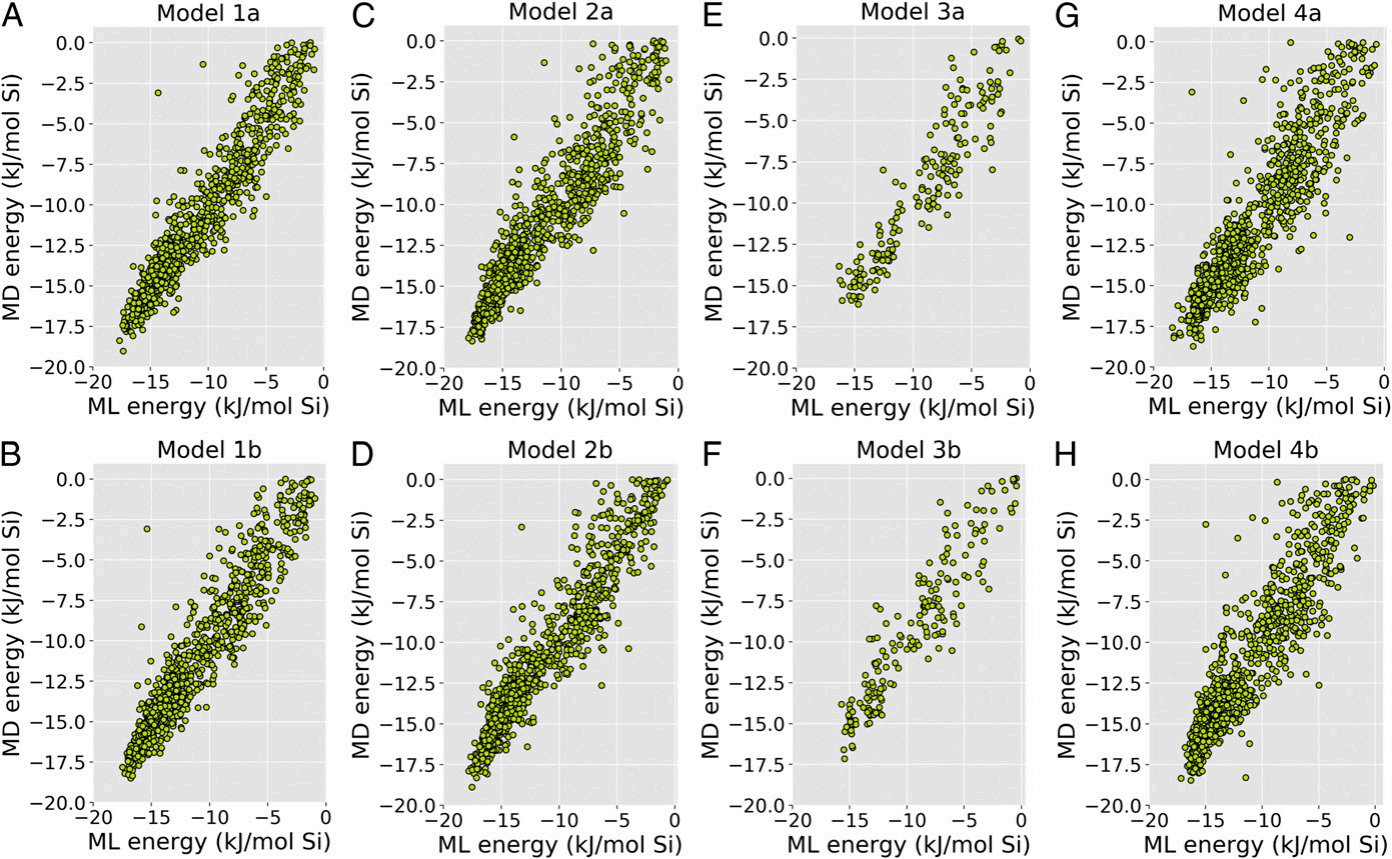
Scatter plots of MD- versus ML-predicted stabilization energies for the OSDAs in the validation set for the eight models (*A*–*H*). Models 1a and 1b were trained on all compounds without weighing. Models 2a and 2b were trained on all compounds with weighing. Compared with models 1a and 1b, models 2a and 2b have better prediction for OSDAs with MD-calculated energy below −15 kJ/mol Si. Models 3a and 3b were trained on charged compounds only without weighing. No charged OSDAs have an MD-calculated energy below −17.5 kJ/mol Si, which limited the ability of the neural network to find favorable OSDAs. Models 4a and 4b used a linear activation function in the output node.

We used ML to relate 3D structure of BEA OSDAs to their stabilization energies. A neural network was trained on the descriptors of molecular structure of OSDAs to predict stabilization energies (see *Methods* and *SI Appendix*, *Materials and Methods*). These descriptors encode the 3D molecular structure by sampling a calculated diffraction pattern. Each scattering parameter, *s*, will produce one intensity, *I*, which is one descriptor. Note that these descriptors are the input to the neural network, not the output. To determine the best-performing set of hyperparameters for the neural network, we tested networks with various values of the maximum scatter parameter ($s_{\text{max}}$), its step size in Fourier space (Δs), and the number of hidden nodes (*h*) in the network. Values for the maximum scatter parameter were 8, 16, 24, and 32 Å, and step size were 0.125, 0.250, 0.500, and 1.000 Å. For each combination of these settings, we randomly choose 80% of the total molecules as a training/test set and set 20% apart for validation (*SI Appendix*, *Materials and Methods* and Fig. S1*B*). The training/test sets were used to train neural networks with increasing number of hidden nodes. The total number of weights in the model depends on the number of input nodes and the number of hidden nodes, the former being determined by the maximal scatter parameter, $s_{\text{max}}$, and its increment, Δs. The highest number of hidden nodes was either 10 or the number of nodes for which the total number of weights was less than the number of molecules in the training set (29). The best model was chosen as the one for which the mean root-mean-square error (RMSE) for test set, $\bar{{\text{RMSE}}_{\text{test}}}$ (*SI Appendix*, Eq. **S5**), was the lowest. This criterion is adopted to avoid overfitting to the training set, as discussed in *SI Appendix*. We trained four variations of the network: a network weighing all MD energies equally, model 1; a network weighing the MD energies below −15 kJ/(mol Si) more, model 2; a network without weighing trained on only the set of charged OSDAs, model 3; and a network without weighing and in which the output layer uses a linear activation function, model 4. A sigmoid activation function was used on the output layer in models 1–3. The results of the exploration of the hyperparameter space are listed in *SI Appendix*, Tables S2–S5. The top two sets of hyperparameters for each model, for which the mean RMSE on the test set, $\bar{{\text{RMSE}}_{\text{test}}}$, was found to be smallest are summarized in Table 1. For these best models, the RMSE for the OSDAs in the validation set ${\text{RMSE}}_{\text{validation}}$ (*SI Appendix*, Eq. **S8**) were calculated. They are listed in column 10 of Table 1. We also calculated the RMSE for the total set of OSDAs in the training plus test set ${\text{RMSE}}_{\text{training}+\text{test}}$ (*SI Appendix*, Eq. **S9**). These are listed in column 9 of Table 1. The validation set has not influenced the hyperparameter selection procedure. The fact that ${\text{RMSE}}_{\text{training}+\text{test}}$ and ${\text{RMSE}}_{\text{validation}}$ are very similar indicates that the neural nets are well trained and not overfit to the train and test set. For reference, the tetraethyl amine OSDA has a stabilization energy of −10 kJ/(mol Si) in zeolite beta A (30). Fig. 1 shows the scatter plots of the MD-calculated and ML-predicted stabilization energies for the OSDAs in the validation set for each of the eight models.

**Table 1.** Top two sets of hyperparameters selected from models 1–4

Model	$s_{\max}$	Δs	Number of intensities	*h*	Total number of weights	$\bar{{\text{RMSE}}_{\text{training}}}$	$\bar{{\text{RMSE}}_{\text{test}}}$	${\text{RMSE}}_{\text{training}+\text{test}}$	${\text{RMSE}}_{\text{validation}}$
1a	24	0.500	49	5	256	1.52 (0.03)	1.79 (0.07)	1.45	1.41
1b	8	0.500	17	8	153	1.59 (0.02)	1.75 (0.06)	1.52	1.47
2a	24	0.500	49	4	205	1.66 (0.04)	1.83 (0.08)	1.50	1.65
2b	8	0.500	17	8	153	1.68 (0.02)	1.84 (0.07)	1.59	1.59
3a	8	0.500	17	2	39	1.61 (0.07)	1.68 (0.14)	1.50	1.64
3b	32	0.500	65	1	68	1.55 (0.04)	1.75 (0.13)	1.55	1.68
4a	32	0.500	65	5	336	1.90 (0.05)	1.92 (0.07)	1.87	1.87
4b	24	0.250	97	2	199	1.91 (0.05)	1.95 (0.09)	1.88	1.89

The $\bar{{\text{RMSE}}_{\text{training}}}$ is defined in *SI Appendix*, Eq. S6, and $\bar{{\text{RMSE}}_{\text{test}}}$ is defined in *SI Appendix*, Eq. S5. The values between brackets are the corresponding SDs. The ${\text{RMSE}}_{\text{training}+\text{test}}$ is defined in *SI Appendix*, Eq. S9, and ${\text{RMSE}}_{\text{validation}}$ is defined in *SI Appendix*, Eq. S8.

Overall, the neural networks were successful at predicting energies of the OSDAs in the validation set. Models 1a and 1b have the best rms error for the validation set, ${\text{RMSE}}_{\text{validation}}$. By introducing weighing in the cost function, models 2a and 2b improve the prediction in the low-energy region below −15 kJ/(mol Si), the region in which OSDAs for BEA are expected to be effective. While this increases the rms error for the validation sets, a modest increase in predictability in this region can be observed in Fig. 1 *C* and *D*. Models 3a and 3b performed equally well (Fig. 1 *E* and *F*). However, no charged OSDAs had MD-calculated energies below −17.5 kJ/mol Si, which limited the ability of the neural network to find favorable OSDAs. Using sigmoid activation, the predicted energies will always be contained in the range of the energies from the training and test set. While this will keep the neural network from erroneously extrapolating to molecules not in this range, it slightly distorts the computed versus predicted relations in Fig. 1. This phenomenon is improved with linear activation, models 4a and 4b, as shown in Fig. 1 *G* and *H*. While the RMSE for model 4 is slightly higher than in the other models, the difference in $\bar{{\text{RMSE}}_{\text{training}}}$ and $\bar{{\text{RMSE}}_{\text{test}}}$ is significantly lower, indicating less overfitting to the training set of this model.

In Table 1 we have a comparison of prediction from single neural networks $\bar{{\text{RMSE}}_{\text{training}}}$ and $\bar{{\text{RMSE}}_{\text{test}}}$ and prediction from averages of 30 neural networks ${\text{RMSE}}_{\text{training}+\text{test}}$ and ${\text{RMSE}}_{\text{validation}}$. The RMSE values of the complete training plus test sets and the validation sets are generally lower than the ones in the training or testing set. This illustrates the capability of the ensemble fitting to improve the models (31): The RMSEs of training and test sets, respectively, are averages taken from multiple single models, *SI Appendix*, Eqs. **S4**–**S6**, while the RMSEs of the complete training plus test sets and the validation sets are from energies predicted from an ensemble of 30 models (*SI Appendix*, Eqs. **S8** and **S9**). However, we also observed that even a single neural network is able to capture most of the predictability of the 30-neural-network ensemble. This result indicates that the predictions of the neural networks are stable to convergence issues and choice of training set.

### In Silico Materials Design.

**Fig. 2.**
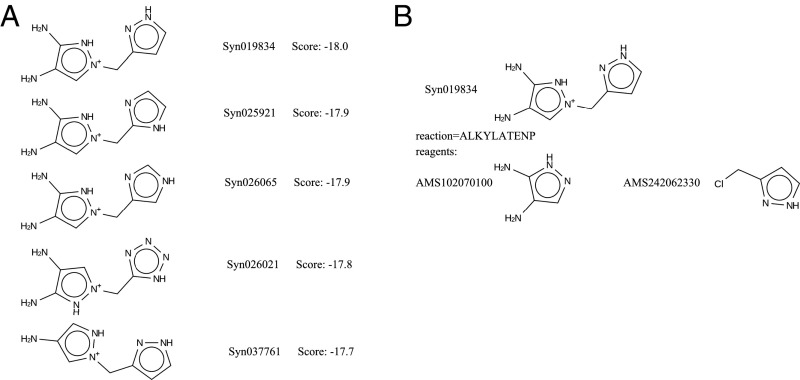
Results for OSDA design using model 1b. (*A*) The top five molecules produced. The molecule scores in this figure are the ML determined binding energy in kJ/(mol Si). (*B*) Proposed synthesis route to the first molecule in the output shown in *A*. The outcome of the synthesis route is listed together with the acronym of the reaction used (ALKYLATENP), as well as the structures and catalog names of the proposed reagents.

We used all eight models in Table 1 in a de novo evolutionary design algorithm program. For each model in Table 1, a total number of 1,000,000 trial molecules were generated by the program and scored using the score vector (see *Methods* and *SI Appendix*, Table S1). Fig. 2*A* shows the top five predicted OSDAs for model 1b. In addition, the synthesis pathway for the top-scoring molecule is shown in Fig. 2*B*. Table 2 lists, for each run, the best OSDA found, with its ML-predicted and MD-calculated stabilization energy, the number of compounds with an ML predicted stabilization energy below −15 kJ/(mol Si), the total number of molecules for which the stabilization energy was actually predicted, and the total number of unique molecules generated. The total number of unique molecules generated during a run is lower than 1,000,000, because molecules may appear, disappear, and then reappear in the population during the course of the genetic algorithm (22). In each run, a large number (∼1,000) of molecules were predicted to have stabilization energies below the threshold of −15 kJ/(mol Si), column 3 in Table 2. The ML- and MD energies of the best-scoring molecules obtained with models 1b, 2a, 2b, and 4b are within 1 kJ from one another; the difference is around 2 kJ for models 1a and 4a. The best-scoring molecules found with models 3a and 3b are identical. Their ML-predicted binding energies are slightly different because the two models have different hyperparameters. The MD-calculated energies differ because of the stochastic nature of the MD procedure. The gaps between the ML and MD energies in models 3a and 3b are larger than for the other models, reflecting the lower prediction precision for these models (see Table 4). The ML method vastly accelerates the energy calculation process. An ML prediction of the stabilization energy of a putative OSDA requires about 28 s of CPU time, whereas an MD energy calculation requires 160 min of CPU time on average.

**Table 2.** Best OSDA found with its ML-predicted and MD-calculated stabilization energy, number of compounds with an ML-predicted stabilization energy below −15 kJ/(mol Si), the total number of molecules for which the stabilization energy was predicted, and the total number of unique molecules generated in each run

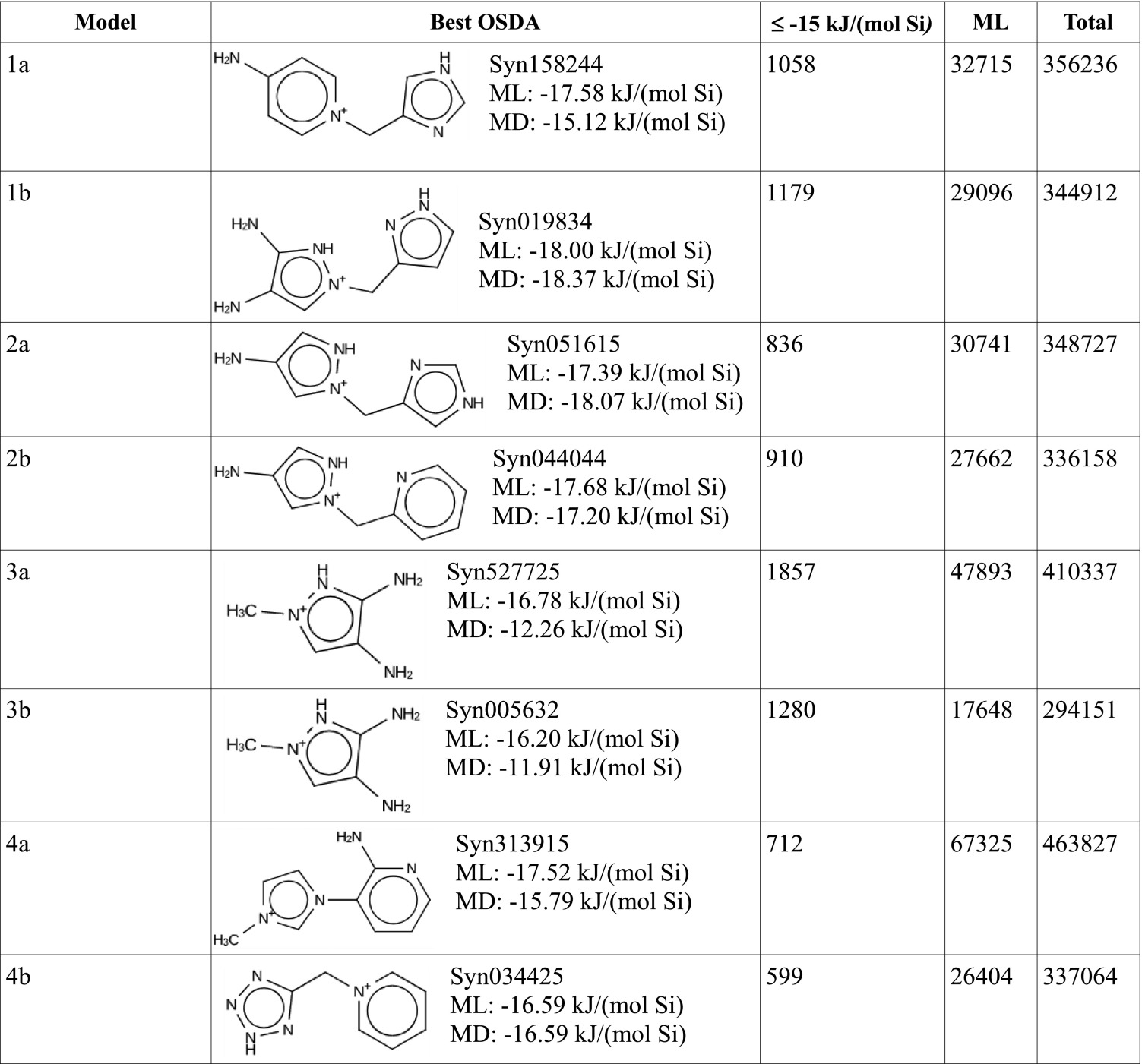

Table 3 shows the cross-section of the putative OSDAs generated in different runs with ML-predicted stabilization energies *E* ≤ −15. kJ/(mol Si). It also lists the number of molecules generated in each run that were also present in the training set. There is considerable overlap between the different runs. This means the different runs have explored overlapping regions in molecular space. As can be seen in column 8 of this table, even some molecules of the training and test sets have been rediscovered. In total, 3,062 highly scoring putative OSDAs have been discovered through our in silico materials design approach. Generally, the goal is to generate as many unique, favorable OSDAs as possible. False positives are not a major concern, since we can easily screen the 3,062 OSDAs with subsequent MD calculation. False negatives are much harder to identify, as it is computationally infeasible to calculate the MD energies for all OSDAs generated by the eight runs.

**Table 3.** Cross-section of the putative OSDAs generated in different runs with ML-predicted stabilization energies *E* ≤ −15. kJ/(mol Si)

Run	1a	1b	2a	2b	3a	3b	4a	4b	In training set
1a	1,058	749	630	560	477	452	497	453	13
1b		1,179	585	691	445	446	402	384	10
2a			836	565	386	374	419	435	11
2b				910	320	312	339	328	7
3a					1,857	1,051	322	254	21
3b						1,280	354	311	12
4a							712	386	17
4b								599	11
Total unique molecules: 3,062									

Column 10 lists the number of molecules generated in one run that are present in the training or validation set.

### Verification.

Ultimately, we validated the materials design on neural network framework by calculating the stabilization energies of the designed OSDAs and comparing them with MD-calculated energies. The goal is to test whether the training, test, and validation sets cover a limited part of the possible chemical space. If so, a neural network trained and validated on such datasets may not necessarily generalize beyond the space on which it was trained. Two possible reasons that can lead to this problem are the possible insufficiency of 3D-MoRSE descriptors to generally describe the 3D molecular structure of the OSDAs, and insufficient complexity of the neural network structure to capture deeper features of the OSDAs’ 3D structure. An in silico materials design run may explore a different chemical space than the training set, and the neural networks may perform poorly and predict inaccurate energies. This concern was tested by comparing the MD energies and ML-predicted energies for the OSDAs.

All molecules generated by in silico materials design and predicted using the ML methods to have a stabilization energy below −14 kJ/(mol Si) were subjected to MD calculation of their stabilization energy for verification. Although it is impractical to calculate the energy by MD for all OSDAs, such calculation on the limited number of predicted OSDAs with stabilization enery below −14 kJ/(mol Si) can give a good estimation of the false negatives. Table 4 lists this measure of false negatives in column 3, with the total number of compounds with ML-predicted energies between −15 and −14 kJ/(mol Si), and the number of compounds among these with MD-calculated energies below −17 kJ/(mol Si). Table 4 also lists the number of compounds with ML-predicted energies below −15 kJ/(mol Si), the number of true positives (TPs), and the prediction precision. Dataset S1 shows all compounds with MD energies below −17 kJ/(mol Si) based upon screening all compounds with predicted ML energies below −14 kJ/(mol Si). In total, there are 469 compounds. This expands upon the 152 compounds with stabilization energy below −17 kJ/(mol Si) that were in our training list of 4,781 compounds.

**Table 4.** The number of compounds with ML-predicted energies below −15 kJ/(mol Si), the number of compounds with ML-predicted energies between −15 and −14 kJ/(mol Si) and among which the number of compounds with MD-calculated energies below −17 kJ/(mol Si), the number of TP, and the prediction precision for the eight in silico materials design runs

Model	$E_{\text{ML}}$≤ −15	−15 < $E_{\text{ML}}$ ≤ −14 ($E_{\text{MD}}$ ≤ −17)	TP (precision)*
1a	1,058 (1,054)^†^	839 (32, 3.8%)	812 (76.7%)
1b	1,179 (1,177)	625 (6, 0.9%)	865 (73.4%)
2a	836 (832)	696 (33, 4.7%)	690 (82.5%)
2b	910 (908)	550 (14, 2.5%)	672 (73.8%)
3a	1,857 (1,840)	915 (60, 6.6%)	727 (39.1%)
3b	1,280 (1,280)	1,204 (104, 8.6%)	660 (51.6%)
4a	712 (695)	827 (34, 4.1%)	538 (75.6%)
4b	599 (599)	805 (57, 7.1%)	484 (80.8%)

* In parentheses is prediction precision, defined as TP/(number with $E_{\text{ML}}$≤ −15) ≡ TP/(TP + FP), where FP is false positive and TP is true positive.

^†^ In parentheses is the number of MD energies, as some MD evaluations failed.

From Table 4 we can see that the false-negative proportion is roughly 5%. The prediction precision is nearly 80% for most models, but 50% for model 3. Among the false positives are some that lie in a different region in the chemical space. For the run with model 1b, for example, we noted that four high-scoring molecules were considerably larger in volume than other molecules in the same and the other runs. They are depicted in *SI Appendix*, Fig. S3, together with their molecular volume and the ML-predicted and MD-verified stabilization energies.

To further investigate the issue of exploring chemical space, we applied a principal coordinate analysis (PCA) analysis (*SI Appendix*, *Materials and Methods*) to the 3D-MoRSE intensities of all molecules generated in run 1b with a predicted stabilization energy to BEA lower than −15 kJ/(mol Si). The scatter plot of the first and second principal components of these molecules is shown in *SI Appendix*, Fig. S4*A*, in which the red dots correspond to the “large” molecules in *SI Appendix*, Fig. S3 and are clearly outliers. *SI Appendix*, Fig. S4*B* shows the scatter plot of the predicted ML stabilization energy versus the molecular volume. The minimal predicted stabilization energy of a molecule follows an approximately parabolic curve with the molecular volume, and the four false-positive hits clearly fall out of this distribution.

A representation of the molecular space explored by the eight in silico runs is presented in *SI Appendix*, Fig. S5*A*. To construct this figure, the 2D Tanimoto fingerprints of the 3,062 unique molecules were generated, and from these a Euclidean distance matrix was computed (*SI Appendix*, *Materials and Methods*). This distance matrix was used to calculate the principal coordinates of each of the 3,062 molecules. The first two principal coordinates are plotted in *SI Appendix*, Fig. S5*A*. The fraction of the variance covered in these two coordinates is 0.20 and 0.10, respectively. Considerable structure is present in this plot, and this can be analyzed in a cursory way by picking representative points and examining the corresponding molecular structures, as is done in *SI Appendix*, Fig. S5*B*. The two large clusters separated by the first principal coordinate distinguish molecules containing aromatic 6-cycle (a through e) and charged pyrazole (f and g) functionalities on the one hand, and charged imidazole functionalities (h through l) on the other hand. Within the two large clusters, smaller subclusters can be discerned that correspond to different molecular scaffolds (*SI Appendix*, Fig. S5*B*). While the specific clustering depends on the choice of the 2D descriptors used for the principal coordinate analysis, this result shows that the in silico material design program produces a variety of molecular scaffolds.

The individual subspaces searched by the eight runs are illustrated in the eight subplots of *SI Appendix*, Fig. S6. In this figure, the molecules generated in each run are represented as green and red dots, and the blue dots correspond to molecules generated in the runs other than the indicated run.

## Conclusions

We have used a data set of 4,781 putative zeolite BEA OSDAs for which the stabilization energies in BEA have been obtained through computationally intensive MD calculation to train ML models for predicting stabilization energies using a neural network. Through exploration of the hyperparameter space we have trained and validated eight models, taking care to strictly separate training and testing sets on one hand, and validation sets on the other hand (32). The molecules generated by the in silico material design fall within the domain of applicability of the ML algorithm. In total we have found 3,062 distinct putative OSDAs for zeolite beta, 469 of which are predicted to be exceptionally stable. We have shown that this protocol enables an effective and computationally tractable search for novel OSDAs.

## Methods

### Neural Network.

The structure of the neural network is shown in *SI Appendix*, Fig. S1*A*. There was one hidden layer between the input and output layer. The input layer consists of structural descriptors *I* of each OSDA obtained through the 3D-MoRSE code (29). The output layer predicts stabilization energies. Sigmoid activation was adopted in the hidden layer. The output layer adopts either sigmoid activation or linear activation depending on the model.

The samples of OSDAs for training, testing, and validating the neural network consist of 4,781 putative BEA OSDAs that we have obtained in our search for OSDAs for pure BEA and chiral BEA zeolite in the past five years. In this search, our procedure consisted of first designing putative small, achiral “monomer” OSDAs and then finding suitable chiral linkers to dimerize these (18). We here use these monomers for training a neural network. To obtain good-scoring monomer OSDAs for BEA we have used three strategies: de novo design, virtual screening, and virtual combinatorial chemistry. A de novo design algorithm (19, 21) was used to generate many putative BEA OSDAs. Analogs of the highest-scoring hits were selected from the available building block databases in eMolecules (https://reaxys.emolecules.com/) and Chemspace (https://chem-space.com/). Finally, we extended this set by generating alkylated derivatives. In this way, we have obtained 4,781 putative BEA OSDAs with predicted stabilization energies between −20 and 0 kJ/(mol Si). These OSDAs consists of a set of 3,875 uncharged molecules and a set of 906 molecules that contain one or several charged N atoms.

### In Silico Materials Design.

The materials design approach is a de novo design program that searches and generates synthesizable molecules with desirable properties. Through a genetic algorithm, this method can search the entire chemical space defined by a list of predefined well-documented organic chemistry reactions and a user-supplied database of commercially available reagents. The output is a set of molecules that score well on the scoring function and their synthesis route.

The score function used for the design of BEA OSDAs is summarized in *SI Appendix*, Table S1. First, it was verified that the molecule to be scored was amenable to molecular mechanics minimization with the force field used. Then the total number of rotatable bonds, the largest number of consecutive sp3–sp3 rotatable bonds, the presence of atoms other than C, N, or H, the presence of triply bonded C, and the ratio of C atoms to charged N atoms were calculated. These properties can all be deduced from the molecular topology and are computationally trivial to obtain. If all of these fell within their respective thresholds, a locally optimal conformation of the molecule was calculated and the molecular volume was obtained. If this fell within its threshold, a conformational search was performed to obtain the global minimal energy conformation of the molecule using GACS. This conformation was used either as a starting point for the MD procedure to obtain the stabilization energy in the zeolite structure, or to calculate the 3D-MoRSE score to be input into the neural network. Here we chose the latter.

The set of reactions used to synthesize virtual molecules presently consists of 100 organic chemistry reactions. The database of reagents we used contains 39,500 commercially available chemicals. To start the run, we randomly selected reactions, reagents, and tree depths to generate the initial population of molecules. Here, tree depth was defined as the number of reactions that take place to form one solution molecule. This depth was usually constrained between 3 and 5. The population size was fixed at $n_{pop}=100$, and every generated molecule was scored. It was possible that some molecules did not pass the scoring filters (*SI Appendix*, Table S1) and therefore did not have the molecular volume or stabilization energy calculated. The population was evolved by applying these reactions and a genetic algorithm search for improved predicted stabilization energies.

Supplementary Materials and Methods, figures, and tables can be found in *SI Appendix*. Detailed materials and methods and discussion of overfitting are available, as well as *SI Appendix*, Figs. S1–S6 and Tables S1–S5, and a .sdf file containing the 469 OSDAs with stabilization energies computed by MD to be below −17 kJ/(mol Si).

## Supplementary Material

Supplementary File

Supplementary File
