# Supplementary material for: Machine-learning approach to the design of OSDAs for zeolite beta
Source: Proc Natl Acad Sci U S A. 2019 Feb 7;116(9):3413–8. doi: 10.1073/pnas.1818763116 (PMC6397530; doi:10.1073/pnas.1818763116)
Supplement: Supplementary File [file pnas.1818763116.sapp.pdf]

PNAS
www.pnas.org

Supplementary Information for

A Machine Learning Approach to the Design of OSDAs for Zeolite Beta

Frits Daeyaert, Fengdan Ye and Michael W. Deem

Prof. Michael W. Deem.
Email: mwdeem@rice.edu

This PDF file includes:

Supplementary text
Materials and Methods
Discussion of Overfitting
Figure S1-S6
Table S1-S5
Caption for database S1
References

Other supplementary materials for this manuscript include the following:

Database S1

www.pnas.org/cgi/doi/10.1073/pnas.1818763116

Supplementary Information Text

1. Materials and Methods

Neural network

We used machine learning to relate three-dimensional structures of BEA OSDAs to their stabilization energies. A neural network was trained on the descriptors of molecular structure of OSDAs to predict stabilization energy. The structure of the neural network is shown in Figure S1A. There was one hidden layer between the input and output layer. Sigmoid activation was adopted on both the hidden layer and the output layer. Given the simple structure of the neural network, it could be trained in a very short time. The trained neural network can predict stabilization energies much faster than can the MD calculation, enabling much more efficient discovery of promising new OSDAs.

The structural descriptors were obtained through the 3D-MoRSE (Molecule Representation of Structures based on Electron diffraction) code (1). This method generates descriptors that encode 3D molecular structure by sampling a calculated diffraction pattern. Each OSDA was optimized geometrically, with its lowest energy geometry chosen using a genetic algorithm for conformation search (GACS). The MoRSE descriptors were calculated on the optimized geometry as follows:

$$I(s) = \sum_{i=2}^{N} \sum_{j=1}^{i-1} A_i A_j \frac{\sin(s r_{ij})}{s r_{ij}} \qquad (S1)$$

where *s* is the scattering parameter, which is sampled on a grid. Each *s* value will produce one intensity, *I*, which is one descriptor. Therefore, fixing the number of s values will fix the number of MoRSE descriptors. Here r_{ij} is the Euclidean distance between *i*th and *j*th atoms, *N* is the total number of atoms, and A_i and A_j are weights for *i*th and *j*th atoms. In our specific model, A_i is chosen as the van der Waals radius for the *i*th atom. Using equation S1, the 3D structure of an OSDA was represented by a list of easily calculable *I* values.

The samples of OSDAs for training, testing, and validating the neural network consist of 4781 putative BEA OSDAs that we have obtained in our search for OSDAs for pure BEA and chiral BEA zeolite in the past five years. In this search, our procedure consisted of first designing putative small, achiral 'monomer' OSDAs and then finding suitable chiral linkers to dimerize these (2). We here use these monomers for training a neural network. To obtain good scoring monomer OSDAs for BEA we have used 3 strategies: *de novo* design, virtual screening, and virtual combinatorial chemistry. A *de novo* design algorithm (3, 4) was used to generate many putative BEA OSDAs. Analogs of the highest scoring hits were selected from the available building block databases in eMolecules (https://reaxys.emolecules.com/) and Chemspace (https://chem-space.com/). Finally, we extended this set by generating alkylated derivatives. In

this way, we have obtained 4781 putative BEA OSDAs with predicted stabilization energies between -20. and 0. kJ/(mol Si). These OSDAs consists of a set of 3875 uncharged molecules and a set of 906 molecules that contain one or several charged N atoms.

To train the neural network, we first shuffled the full set of 4781 samples and randomly selected 20% as the validation set and the remaining 80% as the training and test set (Figure S1B). We normalized the 3D MoRSE intensities so that each descriptor (I value) had a mean of zero and a standard deviation of one across all samples in the training and test set. The normalized I values are denoted as $\tilde{I}$ in Figure S1A. The substracted mean $[\mu_1, \mu_2, \ldots, \mu_p]$ and the original standard deviation $[\sigma_1, \sigma_2, \ldots, \sigma_p]$ were recorded for later use. During training, the stabilization energies calculated from molecular dynamics (MD) were used as the "ground truth" values for each sample. These "ground truth" values were scaled, since sigmoid activation on the output layer predicts values of $\hat{y}$ between 0 and 1, instead of real energy values. The scaling was carried out as follows:

$$y^{(i)} = \frac{E^{(i)} - E_{\min}}{E_{\max} - E_{\min}} \qquad \text{(S2)}$$

where $E^{(i)}$ is the stabilization energy calculated from molecular dynamics on OSDA i, and $E_{\min}$ ($E_{\max}$) is the minimum (maximum) of all MD-calculated stabilization energies of the training and test set. The values of $E_{\min}$ and $E_{\max}$ were recorded for later use. Similar to the normalization of input I values, this scaling was done without the validation set. The idea is that the validation set will serve as the final test of the neural network's performance and is deemed "unseen" throughout the network training and hyperparameter tuning process. We also tested a variation of the neural network design, where the output layer uses a linear activation. In this case, the prediction $\hat{y}$ has no value limit and was a direct prediction of the MD-calculated stabilization energy, $y^{(i)} = E^{(i)}$.

Given a training set of m samples, the cost function is defined using weighted root mean square error (RMS error, or RMSE):

$$\text{RMSE} = \sqrt{\frac{1}{m}\sum_{i=1}^{m} w^{(i)}(\hat{y}^{(i)} - y^{(i)})^2} \qquad \text{(S3)}$$

where $\hat{y}^{(i)}$ is the predicted scaled stabilization energy of OSDA i, $y^{(i)}$ is the scaled MD-calculated stabilization energy of OSDA i, and $w^{(i)}$ is the weight for OSDA i. We experimented with two protocols for the weight $w^{(i)}$. The first protocol was $w^{(i)} \equiv 1$, for all $i = 1,2,\ldots,$ m. Equation S3 is then the regular root mean square error. The second protocol was $w^{(i)} = 10$, if $E^{(i)} \leq -15$ kJ/(mol Si) and $w^{(i)} = 1$ otherwise. OSDAs with energy below -15 kJ/(mol Si) were regarded as good candidates, and we wanted to ensure that the predictions for these OSDAs was as accurate as possible. The factor of ten difference in $w^{(i)}$ ensured that a poor prediction on OSDAs with favorable energies was heavily penalized. The Broyden–Fletcher–

Goldfarb–Shanno (BFGS) algorithm was used to minimize the root mean square error and to obtain the final weights of the neural network.

In the training process, we randomly selected 80% of the training and test set as the training set and the remaining 20% as the test set (Figure S1B). The neural network was trained on the training set. Tunable hyperparameters of the neural network include the maximum s value in equation S1, $s_{\max}$, increment, Δs, and number of nodes, h, in the hidden layer. For each set of hyperparameters, this random sampling and training process was repeated 30 times, producing 30 trained neural networks (NN_1 through NN_{30} in Figure S1B).

To select the best-performing hyperparameters $s_{\max}$, Δs, and *h*, we evaluated the performance of neural networks on test sets. For each of the 30 trained neural networks, we calculated the test set root mean square error:

$$\mathrm{RMSE}_{\mathrm{test},j} = \sqrt{\frac{1}{m_{\mathrm{test},j}} \sum_{i=1}^{m_{\mathrm{test},j}} w^{(i)} (\hat{E}_j^{(i)} - E^{(i)})^2} \qquad \text{(S4)}$$

Where $\mathrm{RMSE}_{\mathrm{test},j}$ is the root mean square error of the *j*th test set and $m_{\mathrm{test},j}$ is the number of samples in this test set. Here $\hat{E}_j^{(i)}$ is the predicted stabilization energy of sample *i* using the *j*th neural network (NN_j), which was trained on the *j*th training set. If linear activation was used on the output layer of the neural networks, we have $\hat{E} = \hat{y}$. Otherwise, an inverse transform of equation S2 was carried out to obtain the unscaled predicted energy: $\hat{E} = E_{\min} + \hat{y}(E_{\max} - E_{\min})$. The overall performance of a given set of hyperparameters is described by the average test RMSE:

$$\overline{\mathrm{RMSE}_{\mathrm{test}}} = \sum_{j=1}^{30} \mathrm{RMSE}_{\mathrm{test},j} \Big/ 30. \qquad \text{(S5)}$$

In the end, we chose the set of hyperparameters with the smallest $\overline{\mathrm{RMSE}_{\mathrm{test}}}$. We also applied a similar measure to the training sets:

$$\overline{\mathrm{RMSE}_{\mathrm{training}}} = \sum_{j=1}^{30} \mathrm{RMSE}_{\mathrm{training},j} \Big/ 30. \qquad \text{(S6)}$$

where $\mathrm{RMSE}_{\mathrm{training},j}$ is the root mean square error of the *j*th training set, defined similar to equation S4. Note that $\mathrm{RMSE}_{\mathrm{training},j}$ is also the cost function being minimized during training of the *j*th network (equation S3).

Finally, we validated the trained neural networks. For each sample in the validation set, we first normalized the *I* values: $\tilde{I}_k = (I_k - \mu_k)/\sigma_k\,, k = 1,2,\ldots,p$. The μ_k and σ_k were the previously recorded mean and standard deviation from the training and test set. The normalized MoRSE descriptors were then input to each of the 30 neural networks with the optimized hyperparameters, producing 30 $\hat{y}$ values for each OSDA in the validation set. Again, if linear activation was used on the output layer of the neural networks, we have $\hat{E} = \hat{y}$. Otherwise, an

inverse transform of equation S2 was carried out to obtain the unscaled predicted energy: $\hat{E} = E_{\min} + \hat{y}(E_{\max} - E_{\min})$. Similar to μ_k and σ_k, $E_{\min}$ and $E_{\max}$ are the previously recorded minimal and maximal energy from the training and test set. For the *i*th OSDA, the average of the 30 $\hat{E}$ values is taken as the final prediction of this OSDA's unscaled stabilization energy:

$$\overline{\hat{E}^{(i)}} = \sum_{j=1}^{30} \hat{E}_j^{(i)}/30 \qquad (S7)$$

where $\hat{E}_j^{(i)}$ is the prediction of OSDA *i* by the *j*th neural network. The root mean square error on the validation set was calculated to evaluate the neural network's performance:

$$\mathrm{RMSE}_{\mathrm{validation}} = \sqrt{\frac{1}{m_{\mathrm{validation}}} \sum_{i=1}^{m_{\mathrm{validation}}} w^{(i)}\left(\overline{\hat{E}^{(i)}} - E^{(i)}\right)^2} \qquad (S8)$$

To compare the neural networks' performance on the validation set and the training and test set, we also calculated the overall root mean square error for the training and test set combined:

$$\mathrm{RMSE}_{\mathrm{training+test}} = \sqrt{\frac{1}{m_{\mathrm{training+test}}} \sum_{i=1}^{m_{\mathrm{training+test}}} w^{(i)}\left(\overline{\hat{E}^{(i)}} - E^{(i)}\right)^2} \qquad (S9)$$

***In silico* materials design**

The validated neural networks were then incorporated into our *in silico* materials design approach to accelerate the stabilization energy calculation. Out materials design approach is a *de novo* design program that searches and generates synthesizable molecules with desirable properties. Through a genetic algorithm, this method searches the chemical space defined by a list of pre-defined well-documented organic chemistry reactions and a user-supplied database of commercially available reagents. The output is a set of molecules that scored well on the scoring function, and their synthesis route.

The score function used for the design of BEA OSDAs is summarized in Table S1. First, it was verified that the molecule to be scored was amenable to molecular mechanics minimization with the force field used. Then the total number of rotatable bonds, the largest number of consecutive sp3-sp3 rotatable bonds, the presence of atoms other than C, N or H, the presence of triply bonded C, and the ratio of C atoms to charged N atoms were calculated. These properties can all be deduced from the molecular topology and are computationally trivial to obtain. If all of these fall within their respective thresholds, a locally optimal conformation of the molecule was calculated and the molecular volume was obtained. If this fell within its threshold, a conformational search was performed to obtain the global minimal energy conformation of the molecule using GACS. This conformation was used either as a starting point for the molecular dynamic procedure to obtain the stabilization energy in the zeolite structure, or to calculate the 3D-MoRSE score to be input into the neural network. Here we chose the latter.

The set of reactions used to synthesize virtual molecules presently consists of 100 organic chemistry reactions. The database of reagents we used contains 39 500 commercially available chemicals. To start the run, we randomly selected reactions, reagents and tree depths to generate the initial population of molecules. Here, tree depth is defined as the number of reactions that take place to form one solution molecule. This depth is usually constrained between 3 and 5. The population size is fixed at $n_{\mathrm{pop}} = 100$, and every generated molecule was scored. It is possible that some molecules do not pass the scoring filters (see Table S1) and therefore do not have the molecular volume or stabilization energy calculated.

This population was sorted using Pareto optimization (5). First, solution molecules that are not dominated by any other solution molecules were placed as in the first Pareto front. With molecules belonging to the first Pareto front removed, the second Pareto front was identified from the remaining population using the same criterion. The third Pareto front was identified with the first and second Pareto front removed. This process continued until all molecules had been ranked. Each Pareto front satisfied the definition that no molecule was dominated by any other molecule in the front. The definition of dominance is as follows:

Molecule i dominates molecule j, if scoring of molecule i is no worse than scoring of molecule j in all objectives, AND scoring of molecule i is strictly better than scoring of molecule j in at least one objective.

For the binary type score "force field compatibility," compatible is better than incompatible. For threshold type score, e.g. molecular volume, a passed score is better than a failed score, and a failed score closer to the threshold is better than a failed score farther from the threshold. Note that there is no superiority between two passed scores with different values. For minimize type score, the smaller value is better than the larger value.

Multiple molecules can exist within each Pareto front. Secondary sorting within each front was carried out based on the order in which molecules were generated and entered the population. As younger molecules have more potential to explore new areas in the chemical search space, they are placed ahead in this sorting.

After the population had been completely sorted, one of the six operators was applied to form a child molecule:

(1) 'add': choose a parent molecule from the current population. Add a randomly chosen reaction step to the reaction path leading to the parent. Additional reagents needed for the new reaction are randomly selected from the reagent database.

(2) 'cut': choose a parent molecule from the current population. Remove the last reaction step from the reaction path leading to the parent.

(3) 'replace random': choose a parent molecule from the current population. Replace one reagent in the reaction path leading to the parent. The new reagent is randomly selected, but must have the required functionality.

(4) ‘replace like’: same as (3), except the new reagent must have a minimal preset chemical similarity to the reagent being replaced.

(5) ‘combine’: choose two parent molecules from the current population. The parents are searched for chemical functionalities that allow them to participate in a reaction. If no such functionality is detected, two other parents are selected. If more than one functionality is detected, one of the allowed reactions is selected at random.

(6) ‘random’: generate a new, random molecule. No parent is needed.

To select parents, we used tournament selection: a set of n_{tour} molecules was randomly selected, and the best-ranking individual was chosen as the parent. If greater than one parent was needed, the randomly selection of n_{tour} molecules was carried out multiple times. Here, we used $n_{\mathrm{tour}} = 2$.

The child molecule was scored and then compared to the worst-ranking molecule in the population. If the child molecule dominated the worst-ranking molecule, the child replaced the worst-ranking molecule, otherwise the child was discarded. If the replacement took place, Pareto optimization was run again to re-rank every molecule in the evolved population. The above ‘select parent – produce child – update population’ step was repeated 1 000 000 times. Putative OSDAs with energies below 0 kJ/(mol Si) were retrained for further analysis.

Principal Component and Principal Coordinate Analysis

To visualize sets of molecules in the reduced space of 3D-Morse intensities, principal component analysis (PCA) was used. The data matrices of intensities were centered and standardized and principal components were extracted using the NIPALS algorithm (6).

To visualize sets of molecules generated by the *in silico* materials design with ML models of differing meta parameters, principal coordinate analysis (PCOA) (7) was applied to distance matrices obtained from the 2-D similarity indices of sets of molecules. Molecules were represented with a binary fingerprint of size 2115 based upon the patterns of up to 4 atoms bound to a central atom, using the MMFF forcefield atom types as atom descriptors. From these fingerprints, a Tanimoto similarity (8) coefficient T was obtained, and 1-T was used as a distance metric for input into the PCOA.

2. Discussion of Overfitting

The $\overline{\mathrm{RMSE}_{\mathrm{test}}}$ is a measure of the quality of the training of the neural network. For most of the results presented, $\overline{\mathrm{RMSE}_{\mathrm{test}}}$ is very close to $\overline{\mathrm{RMSE}_{\mathrm{training}}}$, Table S2-S5, indicating that neural networks were well trained and not particularly overfit to the training set for the sets of hyperparameters examined. Of course, models with the lowest $\overline{\mathrm{RMSE}_{\mathrm{test}}}$ are the best, as it is shown in Figure S2 that the $\overline{\mathrm{RMSE}_{\mathrm{training}}}$ continues to decrease with the number of hidden nodes in the network, whereas there is an optimal number of hidden nodes for $\overline{\mathrm{RMSE}_{\mathrm{test}}}$ due to slight overfitting past that point. The optimal number of hidden nodes for the other models was

determined in the same way. In addition, it is possible that neural networks overfit to the train and test set and perform poorly on the independent validation step. By examining the best models from Table 1 (see main text), we find that $\mathrm{RMSE}_{\mathrm{training+test}}$ and $\mathrm{RMSE}_{\mathrm{validation}}$ are very similar. This result indicates that the neural nets are well trained and not overfit to the train and test set.

Supplementary Figures

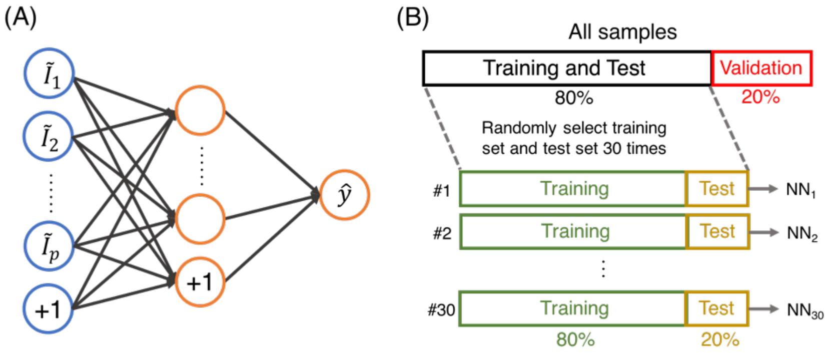

Figure S1. (A) Structure of the neural network. Here $\tilde{\mathbf{I}}$ denotes the normalized MoRSE intensities, and $\hat{\mathbf{y}}$ denotes the predicted scaled stabilization energy. (B) Illustration of the random sampling process. A total of 4781 OSDA samples were shuffled. First, 20% of the samples were randomly selected and set aside as the validation set, and the remaining 80% as the training and test set. Then, 20% of the training and test set was randomly selected as the test set and the remaining 80% as the training set. The process was repeated 30 times, eventually producing 30 trained neural networks.

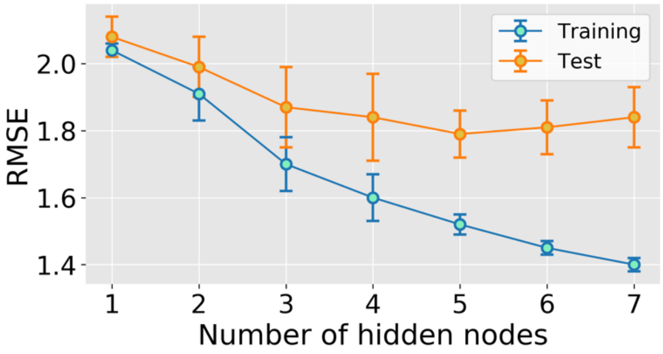

Figure S2. $\overline{RMSE}_{\mathrm{test}}$, and $\overline{RMSE}_{\mathrm{training}}$ as a function of number of hidden nodes in the neural network. The corresponding hyperparameters for this figure are: s_{max}=24, Δs=0.5, h = 1,2,3,…,7 with no weighing and with sigmoid activation. The networks were trained on all compounds, instead of only the charged ones. Note that h=5 corresponds to Model 1a in Table 1. Orange: $\overline{RMSE}_{\mathrm{test}}$, blue: $\overline{RMSE}_{\mathrm{training}}$. The error bars are the standard deviations of RMSE's from 30 NNs of the same set of hyperparameters (see SI Appendix Materials and Methods).

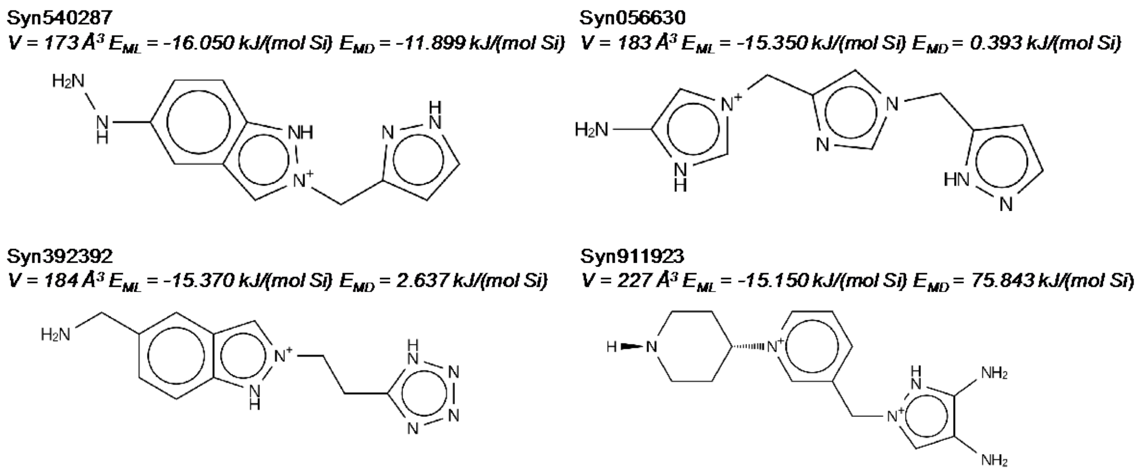

Figure S3. 'Large' false positive OSDAs found in model 1b. Each panel shows one molecule with its name, e.g. Syn540287, volume V, ML predicted stabilization energy E_{ML}, and MD calculated stabilization energy E_{MD}.

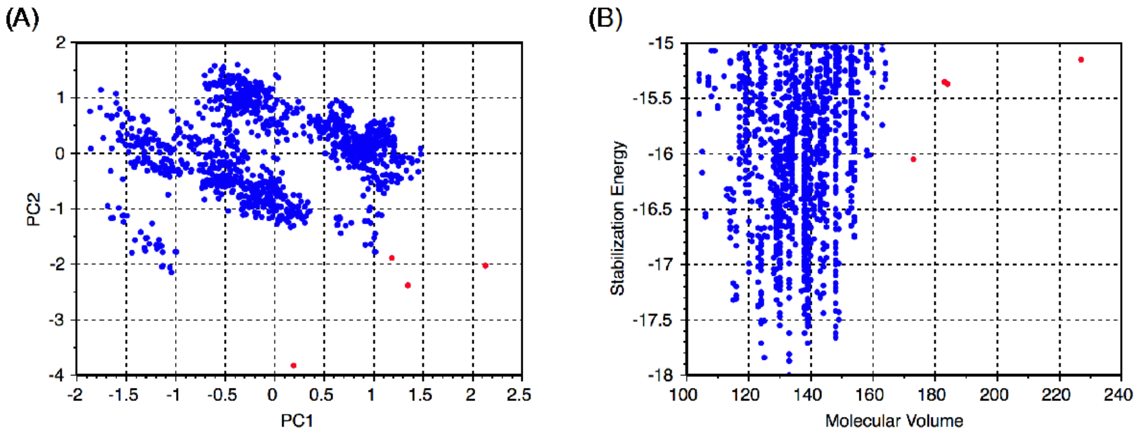

Figure S4. (A) *In silico* materials design run with model 1b: PCA on the 3D-morse vectors of the molecules with a stabilization energy lower than -15 kJ/(mol Si). The red dots correspond to the 'large' molecules. The fraction of the total variance covered by the 2 principal components is 26.8% and 19.9%. (B) *In silico* materials design run with model 1b: ML predicted stabilization energy versus molecular volume. The red dots correspond to the 'large' molecules.

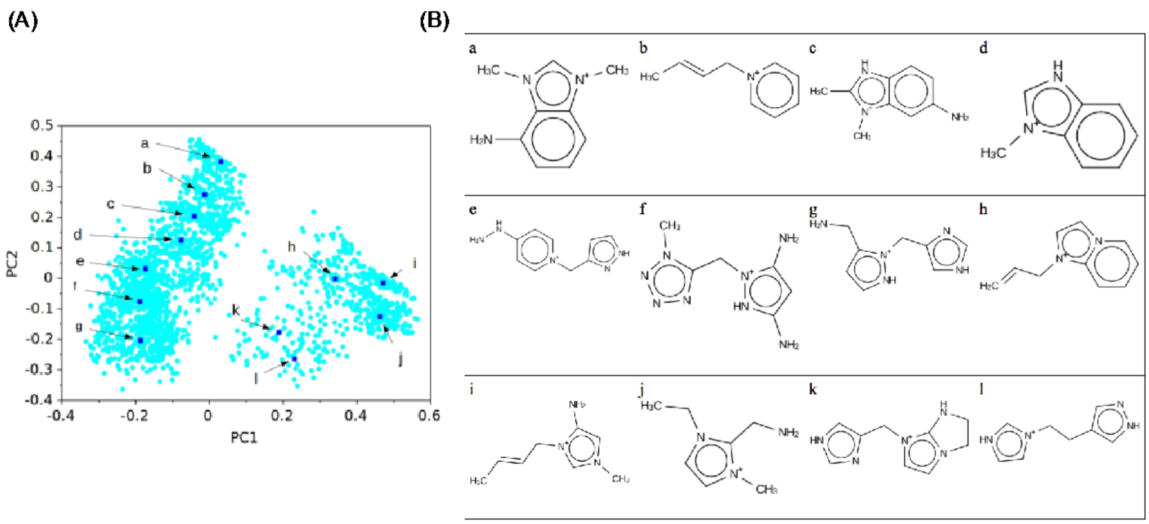

Figure S5. (A) Principal coordinate analysis of 3062 unique putative OSDAs with a stabilization energy in BEA lower than -15 kJ/(mol Si). The x and y axes are the first and second principal coordinates, respectively. (B) Molecules and labels corresponding to the blue squares and labels in A.

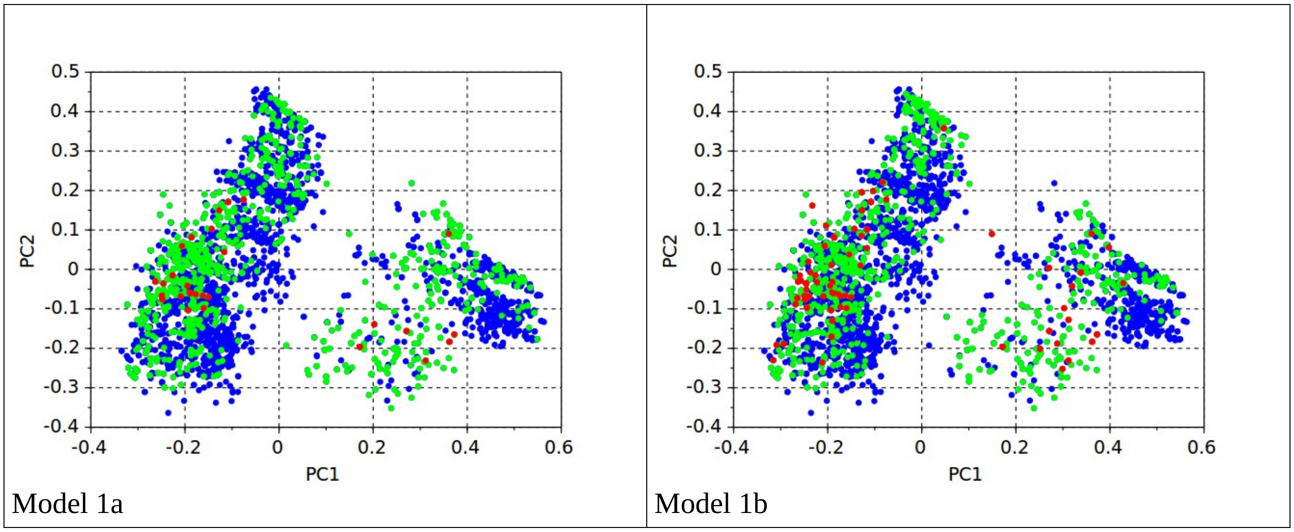

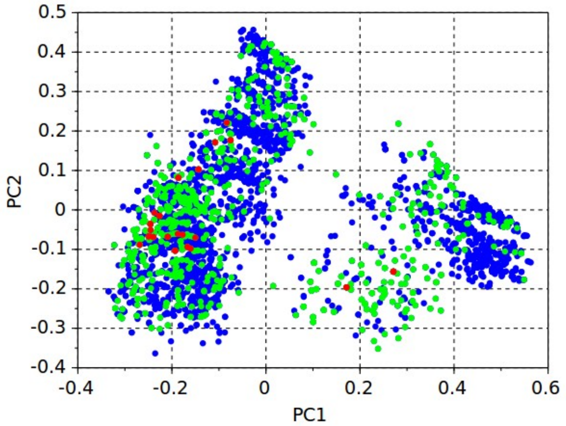

Model 2a

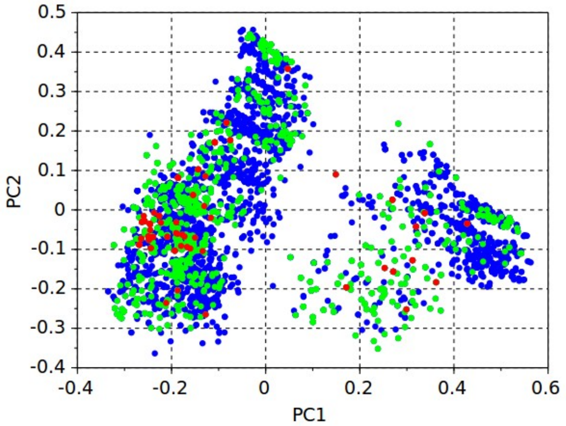

Model 2b

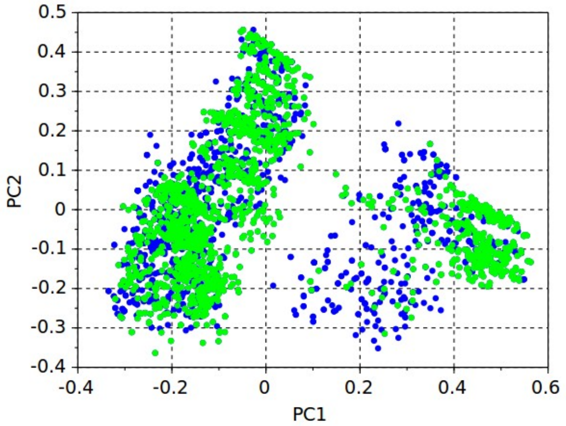

Model 3a

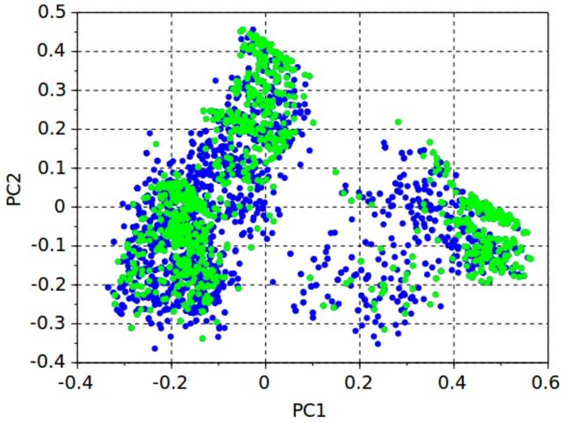

Model 3b

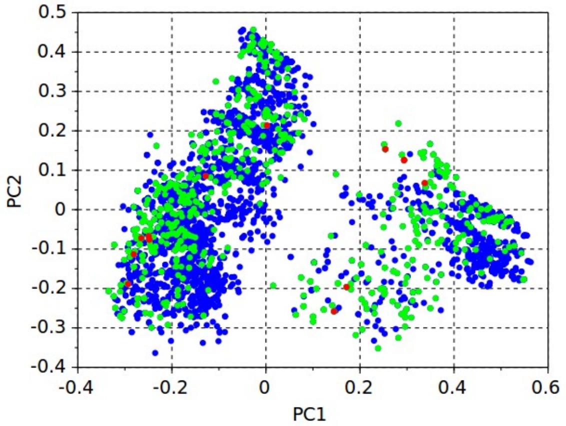

Model 4a

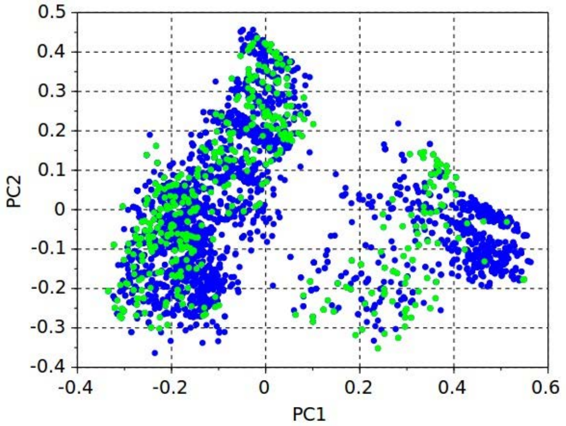

Model 4b

Figure S6. PCOA scatter plots of the molecules with a predicted ML stabilization energy in BEA lower than -15 kJ/(mol Si) generated in the eight *in silico* runs. The green and red dots are the compounds generated in the model indicated at the lower left of each subplot. The red dots have a predicted ML

stabilization energy in BEA lower than -17 kJ/(mol Si). The blue dots correspond to molecules generated in the other seven models.

Supplementary Tables

Table S1. Filter-type score vector comprising the score function for the design of BEA OSDAs.

score description	score type	threshold
force field compatibility	binary	
number of rotatable bonds	≤	6
longest sp3-sp3 chain	≤	3
non -C, -N or -H atoms	≤	0
triply bonded C	≤	0
C to charged N ratio	≤	18
molecular volume	≤	800
Stabilization energy	minimize	

Table S2. Hyperparameter optimization, model based on all compounds without weighing. Here $s_{\max}$ is the maximum scatter parameter, Δs is the step size in Fourier space of the intensities, and h is the number of hidden nodes in the neural network. The $\overline{RMSE}_{\text{training}}$ is defined in equation S6, and $\overline{RMSE}_{\text{test}}$ is defined in equation S5. The values between brackets are the corresponding standard deviations. The two best models are labeled in bold.

$s_{\max}$	Δs	**Number of intensities**	***h***	**Total number of weights**	$\overline{\mathbf{RMSE}}_{\mathbf{training}}$	$\overline{\mathbf{RMSE}}_{\mathbf{test}}$
32	1.000	33	4	141	2.01 (0.03)	2.22 (0.09)
32	0.500	65	3	202	1.67 (0.08)	2.01 (0.11)
32	0.250	129	1	132	1.91 (0.02)	1.87 (0.06)
32	0.125	257	1	260	1.90 (0.02)	2.22 (0.07)
24	1.000	25	5	136	2.03 (0.03)	2.21 (0.09)
**24**	**0.500**	**49**	**5**	**256**	**1.52 (0.03)**	**1.79 (0.07)**
24	0.250	97	1	100	1.96 (0.01)	2.02 (0.06)
24	0.125	193	1	196	1.95 (0.02)	2.05 (0.07)
16	1.000	17	6	115	2.13 (0.02)	2.33 (0.08)
16	0.500	33	4	141	1.66 (0.04)	1.84 (0.08)
16	0.250	65	3	202	1.76 (0.06)	1.95 (0.09)
16	0.125	129	1	132	2.01 (0.02)	2.05 (0.07)
8	1.000	9	8	89	2.45 (0.05)	2.58 (0.07)

s_{max}	Δs	Number of intensities	h	Total number of weights	$\overline{RMSE_{training}}$	$\overline{RMSE_{test}}$
**8**	**0.500**	**17**	**8**	**153**	**1.59 (0.02)**	**1.75 (0.06)**
8	0.250	33	4	141	1.71 (0.03)	1.87 (0.08)
8	0.125	65	4	269	1.72 (0.05)	1.90 (0.11)

Table S3. Hyperparameter optimization, model based on all compounds with weighing. Here $s_{\max}$ is the maximum scatter parameter, Δs is the step size in Fourier space of the intensities, and h is the number of hidden nodes in the neural network. The $\overline{RMSE_{\text{training}}}$ is defined in equation S6, and $\overline{RMSE_{\text{test}}}$ is defined in equation S5. The values between brackets are the corresponding standard deviations. The two best models are labeled in bold.

s_{max}	Δs	Number of intensities	h	Total number of weights	$\overline{RMSE_{training}}$	$\overline{RMSE_{test}}$
32	1.000	33	6	211	2.03 (0.03)	2.34 (0.08)
32	0.500	65	4	269	1.67 (0.06)	1.95 (0.12)
32	0.250	129	2	263	1.79 (0.05)	2.05 (0.12)
32	0.125	257	1	260	2.07 (0.02)	2.19 (0.07)
24	1.000	25	7	190	2.08 (0.03)	2.34 (0.09)
**24**	**0.500**	**49**	**4**	**205**	**1.66 (0.04)**	**1.83 (0.08)**
24	0.250	97	2	199	1.86 (0.07)	2.09 (0.11)
24	0.125	193	1	196	2.12 (0.02)	2.23 (0.07)
16	1.000	17	8	153	2.23 (0.03)	2.42 (0.10)
16	0.500	33	5	176	1.66 (0.03)	1.88 (0.11)
16	0.250	65	3	202	1.83 (0.04)	1.99 (0.07)
16	0.125	129	2	263	2.01 (0.08)	2.15 (0.08)
8	1.000	9	8	89	2.75 (0.05)	2.90 (0.10)
**8**	**0.500**	**17**	**8**	**153**	**1.68 (0.02)**	**1.84 (0.07)**
8	0.250	33	9	316	1.58 (0.02)	1.87 (0.10)
8	0.125	65	4	269	1.83 (0.05)	2.03 (0.08)

Table S4. Hyperparameter optimization, model based on charged compounds only and no weighing. Here $s_{\max}$ is the maximum scatter parameter, Δs is the step size in Fourier space of the intensities, and h is the number of hidden nodes in the neural network. The $\overline{RMSE_{\text{training}}}$ is defined in equation S6, and $\overline{RMSE_{\text{test}}}$ is defined in equation S5. The values between brackets are the corresponding standard deviations. The two best models are labeled in bold.

s_{max}	Δs	Number of intensities	h	Total number of weights	$\overline{RMSE_{training}}$	$\overline{RMSE_{test}}$
32	1.000	33	1	36	1.94 (0.03)	2.02 (0.13)

s_{max}	Δs	**Number of intensities**	h	**Total number of weights**	$\overline{RMSE_{training}}$	$\overline{RMSE_{test}}$
**32**	**0.500**	**65**	**1**	**68**	**1.55 (0.04)**	**1.75 (0.13)**
24	1.000	25	1	28	2.09 (0.03)	2.14 (0.14)
24	0.500	49	1	52	1.60 (0.03)	1.79 (0.13)
16	1.000	17	1	20	2.26 (0.03)	2.33 (0.11)
16	0.500	33	1	36	1.72 (0.03)	1.76 (0.13)
16	0.250	65	1	68	1.64 (0.03)	1.85 (0.13)
8	1.000	9	2	23	2.52 (0.04)	2.63 (0.16)
**8**	**0.500**	**17**	**2**	**39**	**1.61 (0.07)**	**1.68 (0.14)**
8	0.250	33	1	36	1.75 (0.03)	1.89 (0.13)
8	0.125	65	1	68	1.76 (0.04)	1.83 (0.15)

Table S5. Hyperparameter optimization, model with linear activation function for the output node, no weighing. Here $s_{\max}$ is the maximum scatter parameter, Δs is the step size in Fourier space of the intensities, and h is the number of hidden nodes in the neural network. The $\overline{RMSE_{\text{training}}}$ is defined in equation S6, and $\overline{RMSE_{\text{test}}}$ is defined in equation S5. The values between brackets are the corresponding standard deviations. The two best models are labeled in bold.

s_{max}	Δs	**Number of intensities**	h	**Total number of weights**	$\overline{RMSE_{training}}$	$\overline{RMSE_{test}}$
32	1.000	33	9	316	2.18 (0.03)	2.21 (0.07)
32	0.500	65	5	336	1.90 (0.05)	**1.92 (0.07)**
32	0.250	129	2	263	1.89 (0.07)	1.95 (0.09)
32	0.125	257	1	260	2.02 (0.06)	2.05 (0.08)
24	1.000	25	10	271	2.25 (0.04)	2.23 (0.06)
24	0.500	49	5	256	1.94 (0.05)	1.97 (0.08)
**24**	**0.250**	**97**	**2**	**199**	**1.91 (0.05)**	**1.95 (0.09)**
24	0.125	193	1	196	2.03 (0.07)	2.09 (0.08)
16	1.000	17	6	115	2.37 (0.03)	2.39 (0.07)
16	0.500	33	5	176	1.98 (0.04)	2.00 (0.07)
16	0.250	65	2	135	1.96 (0.06)	1.99 (0.08)
16	0.125	129	2	263	1.97 (0.06)	2.03 (0.07)
8	1.000	9	4	45	2.78 (0.03)	2.77 (0.08)
8	0.500	17	2	39	2.14 (0.03)	2.16 (0.06)
8	0.250	33	2	71	2.09 (0.05)	2.09 (0.09)
8	0.125	65	2	135	2.06 (0.03)	2.08 (0.08)

Caption for database S1. All 469 compounds with MD energies below -17 kJ/(mol Si) based upon screening all compounds with predicted ML energies below -14 kJ/(mol Si).

References

1. Schuur JH, Selzer P, & Gasteiger J (1996) The coding of the three-dimensional structure of molecules by molecular transforms and its application to structure-spectra correlations and studies of biological activity. *J Chem Inf Comp Sci* 36(2):334-344.
2. Brand SK, *et al.* (2017) Enantiomerically enriched, polycrystalline molecular sieves. *P Natl Acad Sci USA* 114(20):5101-5106.
3. Daeyaert F & Deem MW (2018) In silico design of chiral dimers to direct the synthesis of a chiral zeolite. *Molecular Physics* 116(21-22):2836-2855.
4. Pophale R, Daeyaert F, & Deem MW (2013) Computational prediction of chemically synthesizable organic structure directing agents for zeolites. *J Mater Chem A* 1(23):6750-6760.
5. Daeyaert F & Deem MW (2017) A Pareto Algorithm for Efficient De Novo Design of Multi-functional Molecules. *Mol Inform* 36(1-2):1600044.
6. Wold H (1966) Estimation of principal components and related models by iterative least squares. *Multivariate Analysis*, ed Krishnaiah PR (Academic Press, New York), pp 391-420.
7. Gower JC (1966) Some Distance Properties of Latent Root and Vector Methods Used in Multivariate Analysis. *Biometrika* 53:325-338.
8. Tanimoto TT (1957) IBM Internal Report.
